# Barriers to supportive care during the Ebola virus disease outbreak in West Africa: Results of a qualitative study

**DOI:** 10.1371/journal.pone.0201091

**Published:** 2018-09-05

**Authors:** Christine Loignon, Elysée Nouvet, François Couturier, Lynda Benhadj, Neill K. J. Adhikari, Srinivas Murthy, Rob A. Fowler, François Lamontagne

**Affiliations:** 1 Faculty of Medicine and Health Sciences, Université de Sherbrooke, Sherbrooke, Canada; 2 School of Health Studies, University of Western Ontario, London, Canada; 3 Department of Critical Care Medicine, Sunnybrook Health Sciences Centre, Interdepartmental Division of Critical Care, University of Toronto, Toronto, Canada; 4 Faculty of Medicine, University of British Columbia, Vancouver, Canada; Mercy Hospital, SIERRA LEONE

## Abstract

**Background:**

During the 2013–2016 West Africa Ebola outbreak, supportive care was the only non-experimental treatment option for patients with Ebola virus disease (EVD). However, providing care that would otherwise be routine for most clinical settings in the context of a highly contagious and lethal pathogen is much more challenging. The objective of this study was to document and deepen understanding of barriers to provision of supportive care in Ebola treatment units (ETUs) as perceived by those involved in care delivery during the outbreak.

**Methods:**

This qualitative study consisted of 29 in-depth semi-structured interviews with stakeholders (decision-makers, physicians, nurses) involved in patient care delivery during the outbreak. Analysis consisted of interview debriefing and team-based transcript coding in NVivo10 software using thematic analysis.

**Findings:**

Participants emphasized three interconnected barriers to providing high-quality supportive care during the outbreak: 1) lack of material and human resources in ETUs; 2) ETU organizational structure limiting the provision of supportive clinical care; and 3) delayed and poorly coordinated policies limiting the effectiveness of global and national responses. Participants also noted the ethical complexities of defining and enacting best clinical practices in low-income countries. They noted tension between, on one hand, scaling up minimal care and investing in clinical care preparedness to a level sustainable in West Africa and, on the other, providing a higher level of supportive care, which in low-resource health systems would require important investments.

**Conclusion:**

Our findings identified potentially modifiable barriers to the delivery of supportive care to patients with EVD in West Africa. Addressing these in the inter-outbreak period will be useful to improve patient care and outcomes during inevitable future outbreaks. Promoting community trust and engagement through long-term capacity building of the healthcare workforce and infrastructure would increase both health system resilience and ability to handle other outbreaks of emerging diseases.

## Introduction

During the 2013–2016 Ebola virus disease (EVD) outbreak in West Africa, supportive care was the only therapeutic option for patients with EVD.[1] Supportive EVD care comprise measures to maintain or improve the physiological status of organ systems and the patient overall. Proven EVD treatments–medications or other treatments specifically targeting the Ebola virus itself or its clinical consequences–were non-existent, and clinical trials evaluating experimental therapies were initiated only late in the outbreak.[2] Mortality from EVD varied between 40% and 70% among 28,616 patients in West Africa,[3] which is considerably higher than the rate of 18.5% observed among 27 patients repatriated for treatment in Europe and the United States.[4] This difference raises questions regarding the prevention of deaths in West Africa if EVD supportive care were to mirror more closely that offered in high-income settings.

Following the West African EVD outbreak, evidence-based guidelines for the provision of supportive care were developed.[5] Recommendations include: 1) administration of oral and, as necessary, intravenous hydration; 2) systematic monitoring of vital signs and volume status; 3) availability of key biochemical testing; 4) adequate staffing ratios; 5) availability of analgesics, including opioids, for pain relief; 6) availability of antibiotics when bacterial infection is suspected; and 7) ability for patients to communicate with relatives to reduce psychological distress. However, the successful future application of these guidelines will require a better understanding of the barriers associated with the provision of supportive EVD care in resource-constrained environments.

Many researchers have focused on the fragility of West African countries’ health systems and the political and socio-political causes of the failure of the global response to the EVD outbreak.[6–8] While the complexity and centrality of social, political, and economic factors in the West African EVD outbreak and its death toll must be acknowledged, it is important to also carefully examine what happened at the level of patient care. There has been scant empirical evidence to document the unprecedented challenges faced by clinicians and decision-makers in Ebola treatment units (ETUs) in Sierra Leone, Guinea, and Liberia.[9,10] Thus, we lack contextual data to better address barriers to the provision of supportive care for EVD patients in resource-constrained environments. The objective of this study was to document and deepen the understanding of barriers related to the provision of supportive care in ETUs as perceived by those involved in delivering or implementing patient care during the outbreak.

## Methods

### Data collection and sampling

We used a descriptive qualitative study approach based on in-depth semi-structured interviews with key informants.[11] This methodological approach was best suited to our research objective of gaining a deep understanding of the complexity of the clinical, public health, and broader socio-political issues that influence the delivery of supportive care to patients during an EVD outbreak. Moreover, this qualitative design, which provides “straight descriptions of phenomena”,[12] is both relevant and advantageous when time is limited and evidence weak on the meaning stakeholders ascribe to events.[12,13] Our study was conducted in accordance with the CERQual criteria.[14]

Using maximum variation and snowball sampling strategies,[15] we purposively selected representatives from relevant stakeholder groups concerned with healthcare delivery during the EVD outbreak in West Africa. These key informants were clinicians and decision-makers who contributed to the international response to the EVD outbreak in West Africa. All interviews were conducted by a qualitative research-trained PhD research assistant (LB). To assist with clinically relevant content, she was accompanied in almost all interviews by a clinician (FL or RF) in our team who had delivered care to patients with EVD during the outbreak. Individual interviews lasting 90–120 minutes were conducted between January and May 2016 by phone or internet-based communication systems following a guide developed according to a predetermined framework. We stopped recruiting upon reaching data saturation, that is, when additional interviews no longer contributed to the understanding of the phenomenon under study.[16] We recorded interviews digitally with the consent of each participant.

### Data analysis

The data were analyzed using an inductive iterative thematic approach. Interviews were transcribed in their original language (English or French) by professionals and coded using software specifically designed for qualitative analyses (NVivo, version 10, QRS International, Doncaster, VIC, Australia). Three bilingual researchers—two experienced qualitative health researchers (EN, CL) and one qualitative-trained PhD research assistant (LB)—conducted the analysis, which included interview debriefing, transcript coding, and data display and interpretation. In debriefings immediately following each interview, researchers reflected on the data collection, summarized findings, identified emerging hypotheses, and prepared subsequent interviews. Two research team members (LB and EN) coded the transcripts independently using NVivo 10 software. They compared their work and reached consensus on a preliminary codebook. We developed codes for themes and sub-themes through independent analysis of transcripts and reinforced them in team discussions. To ensure that subsequent interpretations were grounded in data and not influenced by researchers’ preconceptions, we (CL, LB, EN) used data analysis triangulation and constant comparison to check and validate interpretations^15^ without involving clinician researchers. Finally, a team of three researchers (CL, FC, LB), including a family physician involved in Africa as a clinician and researcher, reviewed the qualitative data report and discussed interpretations regarding the main barriers to optimal care for EVD patients.

### Ethics

Participants were invited through a letter sent by email in English or French, depending on their primary language. Following a positive response, we contacted them by phone to plan an interview and requested an emailed signed consent form prior to the interview. The study protocol and recruitment procedures were approved by the Centre Intégré Universitaire de Santé et des Services Sociaux (CIUSSS) de l’Estrie, in Canada.

## Results

### Description of participants

We interviewed 29 clinicians and decision-makers (eight women, 21 men) involved in delivering care to West African patients during the EVD outbreak. Of these, 28 were clinicians involved in direct clinical care or in organizing the delivery of care to patients in different ETUs during the outbreak. Continents of origin represented in the sample included Australia (1), Africa (7), Europe (9), and North America (12). Participants’ involvement in the outbreak varied and occurred via the affected countries’ ministries of health and governmental (e.g. public health agencies and relevant ministries) and non-governmental (e.g. Doctors Without Borders, Red Cross) organizations responsible for healthcare delivery, and the World Health Organization (Table 1). Seventeen participants had worked exclusively for an NGO during the outbreak, and two had participated in the EVD response as part of both governmental and non-governmental organizations.

**Table 1:** Description of participants (n = 29).

Characteristics	N	%
**Language**		
French	4	14
English	25	86
**Gender**		
Female	8	28
	21	72
**Professional background**		
Nursing	3	10
Medicine	25	86
Other	1	4
**Continent of origin**		
Africa	7	24
Australia	1	4
Europe	9	31
North America	12	41
Type of organization*		
Non-governmental	17	58
Governmental	10	34
Inter-governmental	4	14

* two participants were involved in many types of organizations.

### Perceived barriers to provision of supportive care for patients with EVD in ETUs

Participants reported three main barriers to supportive care for EVD patients in ETUs: 1) lack of material and human resources; 2) organizational structure limiting the provision of supportive clinical care; and 3) delayed and poorly coordinated policies limiting the efficiency of the global and national responses.

### Lack of material and human resources in ETUs

All participating stakeholders highlighted the shortage of material resources in ETUs, especially in the early months of the epidemic.

*So you know*, *the limitations in supplies and medication were mostly around the beginning when we were setting things up*, *and once we were able to establish our supply chain better*, *then we had more of the medication and supplies that we needed*. (AN14)

*We didn’t have pumps* [IV pumps], *we were squeezing the bags of fluids manually*, *so it was very*, *very basic at the time*. (AN24)

Shortages included basic items such as syringes, medicines, and electricity, but also more complex technologies perceived to be crucial, such as diagnostic and monitoring devices.

*Still*, *there were limitations in terms of equipment and laboratory testing that prevented us from being able to provide full supportive care*. (AN12)

*I gave an example of an x-ray*. *In my experience*, *and I’ve seen almost… 600 Ebola patients*, *but I’ve never had an x-ray of an Ebola patient*. (AN 11)

*In West Africa*, *so you just have limitations in general of supportive care*, *there’s no… ventilators*, *there’s no equipment to intubate patients*, *you don’t have dialysis machines*, *you don’t have*, *you know*, *even necessarily*,*… electricity 24/7*, *you don’t have monitoring equipment to monitor the patients carefully in terms of vital signs*. (AN 22)

Several participants noted the lack of adequately trained ETU personnel for specific tasks, such as inserting intravenous cannulas and interpreting lab results.

*They were often really not well trained*, *they had*, *even under normal conditions… difficulties putting in an IV line*, *and how are you going to train to do it safely in… this environment*? *So we had to rapidly increase the staff*, *but the patients were already there*, *so we couldn’t take this staff for one week to train them quietly somewhere*, *they had to be with patients*. (AN13)

*Obviously there is the issue of human resources to do laboratory testing in the field; that level of expertise isn’t [available] far and wide*, *but some of these technologies are relatively basic*, *and so environment becomes the overarching limiting factor in the [ETU] unit*. (AN07)

In contrast, those who were involved for several months observed that patient outcomes improved when resources became more widely available in later stages of the epidemic.

*I saw a drastic change from oral rehydration solutions to IVs*. *There was massive improvement in the… [number] of survivors*. (AN 20)

*By the time we built our lab capacity*, *we were able to know whether the CT [cycle threshold*, *which is inversely proportional to viral load] values were increasing or decreasing*, *and that also helped our clinical management*. (AN 22)

Such observations support the idea that better access to basic and more complex material resources in ETUs, such as diagnostic and monitoring equipment, might improve supportive care implementation and patient outcomes.

### ETU organizational structure limiting the provision of supportive clinical care

Participants perceived that certain ETU characteristics interfered with the provision of supportive care and caused disparities in the quality of care across ETUs. For instance, the lack of clinical protocols hindered the standardization of best practices even within organizations.

*There shouldn’t be any differences from one centre to another*. *If there’s the capacity to be a centre*, *then in the same country*, *both treatment centres must have the same capacities*. (FR01)

*Not saying*, *well*, *if you work for X organization you’re going to do this*, *and if you’re deployed by X organization you’re going to do that*, *and if you work for this NGO*, *it’s going to be different*. *Not that there are drastic differences*, *but there are some differences*, *so if we could move forward to have a unified approach in terms of a clinical care protocol…*. (AN03)

*During the outbreak*, *where some organizations decided that they would not provide IV… given anything that had to do with needles*, *they wouldn’t give IVs*, *wouldn’t give IV fluids*, *or give injectables*. *So given that’s considered to be the cornerstone of management of Ebola patients*, *that’s a really difficult thing to swallow*, *to say that the only thing that you can do to save this patient’s life*, *you’re not allowed to do*. *Having these decisions without really a lot of evidence and just using what we know to that point to inform that decision*, *I think is regrettable*. (AN17)

ETU structures were sometimes perceived as contributing to patient isolation and dehumanization of care. Some participants expressed indignation over inhumane ETU environments.

*But that’s crazy*, *because then you’re leaving people for four hours*, *you don’t know what’s happening*. *People are dying*, *people are having seizures*. (AN05)

*… [it’s important to] humanly allow them to still contact their family*, *they can still see their family through the fence*, *that they still have contact*. *We have to humanize the approach to Ebola*. (AN04)

Certain participants perceived that decisions regarding the delivery of supportive care were either arbitrary or dependent upon the leadership of individuals who were only involved for limited and variable periods.

*Because especially [with this NGO] everybody has their own opinion*, *everybody thinks they know the right thing to do*. *And there are protocols in place*, *but everybody feels they know something better*. *So everything gets changed every six weeks*. (AN05)

Variations across ETUs were also attributed to deficiencies in communication between ETUs and data collection processes, which in turn suggested insufficient planning and preparation.

*We need to plan early*. *And we need to know our groups*, *and we need to work with the people who know each other*. *To me that’s very*, *very important*, *so that we speak… the same language*. (AN11)

*The information management was not great*. *There should be a minimum standard in terms of information and collecting appropriate clinical data on patients*, *as well in terms of treatment they received*, *how sick they were*, *how did they improve*. (AN12)

Participants reported that some EVD patients did not receive adequate care and that the shortage of trained staff in some ETUs due to lack of planning or weak leadership contributed to poor performance.

*[*…*] but I think hierarchy was also unclear*, *who’s responsible for what*. *Otherwise*, *what I’ve seen in some other centres*, *I think it’s really… yeah*, *that can be a major problem*. *I’m not sure it’s a barrier*, *but it makes it very dangerous to work… it’s a dangerous thing to do*, *right*? *If it’s not clear who’s responsible for what…* (AN13)

In summary, our participants discussed organizational barriers, such as lack of standardization of care and deficient management structures in ETUs, that prevented patients with EVD from receiving adequate supportive and patient-centred care during the outbreak.

### Delayed and poorly coordinated policies limiting the efficiency of the global and national responses

Participants recognized that the magnitude of the EVD outbreak presented unprecedented challenges for healthcare organizations as well as governmental and non-governmental organizations. However, participants ascribed poor outcomes to belated and poorly coordinated outbreak response policies. Frustration over the national and international emergency response to the EVD outbreak was a recurring theme.

*The system barriers [led to] the failure to mobilize enough treatment teams in the first three months [out] of fear*, *but [they] just sent their teams*. *Even though they had thousands of [clinicians] to send*, *they didn’t have organizations capable of deploying outbreak-ready clinical teams*. *That is the number one barrier for the global health system*. (AN04)

*We need to react sooner*. *Obviously one of the big issues was that the humanitarian community was late to the game in this*. *Ebola started in December 2013*, *and really nobody got there until the next summer*, *if that*. (AN12)

According to participants, these delays were mainly attributable to insufficient political leadership and to a lack of early epidemiological surveillance. Participants believed these resulted in late presentations of EVD patients and avoidable cross-contamination between suspected and confirmed EVD-infected patients in overcrowded ETUs.

…*the big human resources benchmarks*, *how fast we can mobilize in the sub-region or region*, *global infectious disease clinical response teams*. (AN04)

Another issue was related to the way patient data management was set up regionally, especially the lack of a consistent approach to recording patient data and having no system in place to share patient information between facilities.

*It was impossible to keep track of patients*. (AN12)

*One thing that we struggled with a lot at the beginning was the medical data*. *The transfer of data from the inside to outside*, *when you’re not supposed to carry stuff*. (AN 13)

*We received patients from holding centres*, *but without ever having information on treatment they had received and the tests they had done*. *So that*, *definitely*, *[is a problem] in terms of the bigger picture of information transfer*. *All these ETCs [ETUs] should be operating like hospitals*. *So you never really admit someone from another hospital without some transfer documents*, *so some information*. *And yet in the ETC we did that a lot*, *where we had no idea what treatment they received*. (AN15)

On another level, deficiencies were noted in communications between health ministries, NGOs, the World Health Organization, other international organizations, ETUs managed by health ministries and NGOs, and local populations.

*If you can’t manage [for] every single clinician to be able to speak*, *at least have good [connections with] the local community that facilitate good communication and early presentation of patients and therefore*, *of course*, *better survival rates*. (AN04)

All participants expressed the need to work closely with national and subnational levels, as well as community representatives, and for any EVD response to take seriously the importance of good community relations. Community engagement was understood as important to patient outcomes, in that misunderstanding or mistrust of ETUs could result in individuals presenting late for care—when the disease was already advanced—or potentially not presenting at all.

*If we weren’t able to establish trust*, *we weren’t able to access the people who needed care*. *That was the first big thing that we had*. *We realized that our main objective at the beginning of the outbreak was to develop a sense of trust within the community*. (AN16)

*There’s so much stuff we need to be doing in the communities that we just aren’t… There are very few organizations doing adequate levels of community involvement*. (AN02)

In summary, participants identified numerous flaws in outbreak response policies, including a delayed international response, lack of communication and data-sharing among organizations, and inadequate community engagement.

### Provision of supportive care for patients with EVD in West Africa: an ethical dilemma

As shown in Table 2, participants enumerated multiple barriers that, in their view, hindered the provision of care to patients in West African ETUs.

**Table 2:** Barriers to delivery of supportive care for patients with EVD.

• Insufficient resources to provide patient-centred care, including pain management and basic nursing care
• Insufficient resources to implement basic supportive care: IV fluids, antimalarials and antibiotics for concomitant infections, laboratory facilities for electrolytes monitoring
• Lack of rapid diagnostic testing
• Absence of resources to provide advanced supportive care: no radiology, dialysis, or mechanical ventilation
• No ability to maintain adequate medical records
• Insufficient public health surveillance to facilitate early presentation to ETU
• Lack of communication between ETU patients and their families
• Shortage of staff, and unpaid local staff
• Lack of transparency from ETUs, with resulting lack of confidence among the population leading to late presentation

Participants conveyed a sense of powerlessness and indignation associated with inability to deliver medical interventions they felt to be both feasible and effective.

*[…] although it was a crisis situation*, *I felt*, *even with proper blood testing*, *we could’ve been far more sophisticated*. *It’s outrageous that Africa doesn’t have labs*, *and they’ve tried to have labs*, *but they’ve never had funding*. *Ebola would have been diagnosed way earlier*. (AN05)

Furthermore, our data highlight a moral dilemma expressed by our participants as to what they perceived should be done, from an ethical standpoint, to provide supportive care for EVD patients in West Africa. This ethical dilemma can be described as one between pragmatist and interventionist perspectives. The pragmatist perspective emphasizes public health and structural causes of barriers to the provision of care to EVD patients, while the interventionist perspective refers to barriers impeding the best evidence-based individual medical treatment. Even when participants recognized that the care provided to EVD patients was suboptimal, many of them questioned the ethics of enacting higher levels of supportive care than those normally provided and achievable in the affected countries, given the severe under-funding and fragility of their health systems.

Participants holding a more pragmatic perspective typically had extensive experience of similar public health emergencies. They emphasized the limited resources in West Africa and the fact that endemic health system failures preceded the EVD outbreak.

*Patients who were treated in developed settings had a lower mortality than those treated in West Africa because of the standard of care being provided*. *The settings they had access to in basic treatments*, *analysis*, *experimental therapy and things like this*. *That’s true for every disease you can think of*. *Every disease is treated better in more developed settings [such as] Western settings*, *than in Liberia*, *for example*. (AN11)

*The lack of resources in the various sub-Saharan African structures*. *That is the main obstacle; it’s just that the resources are not there*. (FR01)

Participants often pointed out that the lack of a well-trained and well-paid workforce in West Africa before the outbreak was an important factor that facilitated a lower standard of care compared to that in high-income country ETUs. They tended to be less in favour of scaling up supportive EVD care, given the time and significant investments that would be needed in the training institutions of the affected countries to render such care sustainable in the long-term.

*I think that that’s one of the things that is a big barrier… Creating that labour force really will require a significant investment in the institutions that [would] produce sophisticated*, *mature biomedical researchers and public health experts and clinicians in West Africa*. *And so that’s not done through going for a three-day workshop and things like that*. *So… the long-term approach… could be considered as one of the potential obstacles*. (AN03)

In contrast, medical specialists with more limited experience in global health were more inclined to favour an interventionist approach. These participants more often stressed the importance of clinical guidelines for supportive EVD care and advocated for quality of care benchmarking measures in spite of limited resources.

*It should’ve been high priority overnight to have labs [so] that we could do more testing*. *To me that was appalling*, *too*, *that the doctors were blind… in a way that [they] didn’t have to be*, *given the… I think it’s called the i-STAT*, *is it*? *We didn’t have one*, *I think maybe you had one in Guinea*, *but there’s so much you can do with just that knowledge*. (AN05)

*The obvious source of deficiency in ETUs*, *which you would have heard from absolutely everybody*, *was the inability to monitor fluids and electrolytes in a way that we can [use] and [to have adequate] resources for each setting*. *And that makes fluid replacement… and supportive care incredibly challenging without the appropriate biochemistry*, *for example*, *to back it up*. (AN07)

Participants reflected that, over the duration of outbreak, knowledge of optimal clinical care for EVD improved, which suggested the feasibility of providing progressively more advanced treatments despite the limited availability of resources.

*And we've seen that over the course of the outbreak in west Africa… the care that was provided was*, *well*, *more robust*, *and in the setting of more robust care being provided*, *the mortality rate went from something like 60–70% down to 30%*. (AN18)

*March–April [2015]*, *we were really starting to be able to do some investigations and to actually figure out what was wrong with our patients*, *and treat what was wrong*, *you know*, *like a potassium imbalance or… Like*, *we just started to know more what’s going on*. *At the very beginning*, *we were just flailing blindly*, *doing anything we possibly could to help*. (AN02)

In summary, our participants revealed how the clinical and organizational barriers to supportive care for EVD patients were intertwined, resulting in EVD patients receiving suboptimal care during the EVD outbreak. Furthermore, decision-makers and clinicians who participated in this study considered that the human, material, and organizational barriers were reinforced by health systems factors, such as an unprepared workforce and insufficient resources to provide even basic healthcare during routine non-outbreak conditions.

## Discussion

To our knowledge, this is the first qualitative study on barriers to supportive EVD care in an outbreak setting that included national and international clinicians and decision-makers closely involved in the provision of care to patients with EVD. According to our interviews of 29 clinicians and decision-makers in the 2013–2016 EVD outbreak, barriers to supportive care consisted of: 1) a lack of material and human resources in ETUs; 2) ETU organizational structures limiting the provision of clinical care; and 3) delayed and poorly coordinated policies limiting the efficiency of the global and national responses. Our findings contribute to a large body of literature on this outbreak and corroborate recent scientific literature that has highlighted the lack of resources and the limited preparation of clinical teams to face the challenges of a large outbreak in West Africa. Our contribution further clarifies *why* and *how* patients with EVD in West Africa did not receive supportive care in 2013–2016.

All participants lamented the low level of care provided in many ETUs. Their accounts revealed that the provision of care to patients was constrained by: the scarcity of trained local physicians, nurses, and allied health professionals; the unreliable access to water, sanitation, hygiene, and electricity; the absence of basic laboratory and monitoring capacities; and the lack of medication and intravenous fluids. Global inequities and resource-constrained national health systems are well-known factors contributing to the emergence and course of outbreaks.[7,17–19] Scientists and bioethicists have highlighted disparities and ethical issues, including “therapeutic nihilism”, related to experimental and standard medical treatments in outbreak conditions.[6,10,20–23] Inadequate outbreak control policies that resulted in delayed international response and poor community engagement were identified as factors contributing to inadequate clinical care.

Importantly, the barriers to high-quality clinical care we identified do not appear to be insurmountable in future outbreaks, as exemplified by the development of evidence-based guidelines for supportive care in ETUs [5] and by the response to the current, albeit smaller, outbreak in DR Congo. First, in future outbreaks, ETUs can be equipped more fully from the outset with diagnostic and supportive care capacity. In the case of EVD, and as outlined in recent recommended guidelines,[5] this must include the option of providing intravenous fluids and pain relief for all patients who require and accept such measures, which involves more monitoring and more diagnostics. Such essential measures are not easy to guarantee in the context of chronically underfunded health systems, which are the norm in low-income countries where EVD presents the highest risks, yet such measures are not impossible.[24]

Second, improving communication and coordination not only among organizations and ETUs, but also with populations in the event of an outbreak, is feasible. Indeed, providing high-quality clinical care is compatible with a community-based care approach, “two-way community engagement”,[25] and community-led social mobilization, as supported by the Ebola Response Anthropology Platform.[26, 27] Active community engagement in acute and public health care—a basic ethical imperative during outbreaks—if continued in non-outbreak periods, would build trust, facilitate contact tracing, and promote early presentation for care in symptomatic individuals.[28] The EVD outbreak in West Africa has created an opportunity to develop mechanisms to involve the community in future outbreaks and to strengthen the health system for routine non-outbreak care.

An unanswered question is whether sustained long-term funding for an international and national surge capacity of healthcare workers and material resources would actually address barriers to supportive care in ETUs. This question lies at the root of the ethical dilemma that emerged among our participants as they were asked about how to sustain best clinical practices for supportive care to EVD patients. Most participants in this study believed that a larger and more coordinated early response, with more personnel and better diagnostic and clinical resources, would have improved clinical outcomes for patients and decreased mortality. Such assessments are consistent with widely circulating explanatory narratives for “what went wrong” during the West African epidemic—narratives foregrounding structurally weak and overwhelmed local health systems and the morally troubling failure of the international community to do more sooner.[24, 29]

A minority of participants, on the other hand, expressed uncertainty about whether it was realistic, or even ethical, for an international humanitarian healthcare response to try to achieve the same standards of supportive care in West African ETUs as in high-income countries. In the chronically under-resourced healthcare context of West Africa,[30–31] striving for “best practice” in ETUs based on high-income country standards struck a number of our respondents as unrealistic. Interestingly, those participants’ views were more aligned with the importance of global governance and sustainability, both of which require a systemic approach, as pointed out by the Independent Panel on the Global Response to Ebola.[32]

Finally, given the presence of animal reservoirs and the pattern of outbreaks observed in Uganda and the Democratic Republic of the Congo, new outbreaks may well recur in West Africa.[33] This possibility underscores the importance of early warning systems and has prompted numerous calls to strengthen inter-outbreak diagnostic capacity and early outbreak responses at local, national, and international levels. It has also led to calls for major international investment in the region to help build robust, responsive, trustworthy, and efficient health systems.[8, 24, 25, 28, 34] This is no easy task but is the best insurance against another uncontained outbreak.

### Methodological considerations, strengths, and limitations of the study

This qualitative study addresses an important knowledge gap in the fields of global health, equity, and outbreak preparedness. The methodology, including sampling strategy, interview guide, and analysis framework, was carefully planned before interviews were conducted. The use of maximum variation sampling increases the transferability of the results. Nonetheless, we acknowledge the following limitations. First, we interviewed a small number of stakeholders, of whom a minority were from Africa and a few from other regions outside North America and Europe; however, their backgrounds reflected a wide spectrum of expertise and outbreak experience in West Africa. Moreover, we attained data saturation, which is a recognized criterion of rigour in qualitative research. In contrast to quantitative analyses, qualitative research often requires fewer participants but allows for more complex, in-depth analyses of individuals’ perspectives and opinions.[35] Second, the sampling frame is at risk for selection bias. Using a snowball sampling approach, we aimed to reduce this risk by asking participants of different clinical and national backgrounds and opinions for the names of additional potential participants. We also attempted to adhere to the principle of internal diversification [15] by inviting decision-makers, health coordinators, and clinicians with diverse and complementary perspectives. Even so, few participants were nurses and government representatives, suggesting possible residual selection bias. Third, we did not interview EVD survivors, their relatives, or community members affected by EVD. Although their perspectives would be crucial to evaluate the perceived quality of care during the outbreak, this is an objective for a future study. Finally, our research team includes clinicians involved with care delivery during the outbreak. Recognizing that this constituted a potential conflict of interest, those co-authors of this article were not interviewed. Although they contributed to the design and methods of the study and to the clinical interpretation, they were not involved in interpreting the transcripts. Qualitative health researcher co-authors were responsible for the methodology and involved in each step of the coding and interpretation of data.

### Implications for practice

These study results may help governmental and non-governmental planners, decision-makers, and clinicians to better anticipate the barriers to providing best clinical care to patients during an EVD outbreak and offer potential solutions to these barriers. Many barriers were due to constrained healthcare resources. In general, preparedness for future EVD outbreaks in resource-constrained settings may benefit from proactive mitigation of these barriers. Having larger, better prepared, and appropriately equipped local and international medical teams will help improve access to supportive care and produce better outcomes for patients.

## Conclusion

Our findings identified modifiable barriers to the delivery of supportive care to patients with EVD in the West African context. Addressing these barriers in the inter-outbreak period will be useful in establishing health systems that will improve patient care and outcomes during inevitable future outbreaks. Promoting community trust and engagement through long-term capacity building of the healthcare workforce and infrastructure would increase health system resilience and ability to handle other outbreaks of emerging diseases.

## Supporting information

S1 File Interview Guides. (DOCX)
