# Supplementary material for: Barriers to supportive care during the Ebola virus disease outbreak in West Africa: Results of a qualitative study
Source: PLoS One. 2018 Sep 5;13(9):e0201091. doi: 10.1371/journal.pone.0201091 (PMC6124726; doi:10.1371/journal.pone.0201091)
Supplement: S1 File — (DOCX) [file pone.0201091.s001.docx]

**Section 1. Introduction and exploration of adequacy of supportive care measures**

1. Could you describe to us your expertise and explain your role during the last Ebola outbreak (EO)?
2. According to your expertise and experience during the EO, how would you describe the clinical management of patients admitted to Ebola Treatment Units (ETUs)? Did you note any changes over time?
3. Is it still possible to improve clinical management of patients admitted within ETUs? How?

**Section 2. Barriers influencing the delivery of supportive care**

1. What, in your opinion, are the main barriers hindering the delivery of optimal supportive care measures to patients admitted within ETUs?
2. How are they related to material resources, supplies and technology available within ETUs?
3. How are they related to human resources for the clinical managment of patients admitted within ETUs?
4. How are they related to the organizational structure of clinical management? Who is responsible for decisions regarding the clinical protocols? How did information flow within and between organizations?
5. Could you remember and share with us a situation that illustrates how some barriers may have hindered the delivery of supportive care?

**Section 3 Perceptions of the facilitators**

1. What, in your opinion, are the main facilitators of optimal supportive care measures for patients within ETUs?
2. How are they related to material resources, supplies and technology? Could you explain?
3. How are they related to human resources for the clinical management of patients admitted within ETUs?
4. How are they related to the organizational structure of clinical management?
5. Could you remember and share with us a situation that illustrates how some facilitators may have improved the delivery of supportive care?

**Section 4 Benchmarks measures**

1. Are there benchmark measures that may help ascertain whether adequate care is provided to patients admitted in ETUs?
2. In your view, what is the strict minimum that must be provided to patients admitted to ETUs?

**Section 5 Solutions, recommandations**

1. What potential solutions would help overcome the barriers that hinder optimal clinical management within ETUs?
2. What are your recommendations concerning the planning, preparation and delivery of care for patients admitted to ETUs?

**Section 5. Conclusions**

1. Is there anything that we have not discussed and which seems relevant to the issue of clinical management within ETUs?

**Section 1. Introduction et exploration du niveau des mesures de soins de soutien**

1. Pourriez-vous nous décrire votre expertise et expliquer votre rôle au cours de la dernière épidémie du virus Ebola?
2. Selon votre expertise et expérience au cours de la dernière épidémie du virus Ebola, comment décririez-vous la prise en charge des patients admis dans les unités de traitement du virus Ebola? Avez-vous noté des changements au fil du temps?
3. Pensez vous qu’il est possible d'améliorer la prise en charge clinique des patients admis dans les unités de traitement du virus Ebola? Comment?

**Section 2. Obstacles influençant la prestation des soins de soutien**

1. Quels sont, à votre avis, les principaux obstacles qui entravent la prestation de soins aux patients admis dans les unités de traitement du virus Ebola?
2. Comment sont-ils liés aux ressources matérielles, ressources humaines ou structures organisationnelles aux fournitures et aux technologies disponibles au sein des unités de traitement du virus Ebola?
3. Comment sont-ils liés aux ressources humaines pour la gestion clinique des patients admis dans les unités de traitement du virus Ebola?
4. Comment sont-ils liés à la structure organisationnelle de la gestion clinique? Qui est responsable des décisions concernant les protocoles cliniques? Comment l'information se transmettait à l'intérieur de l’organisation et entre les organisations?
5. Pouvez-vous penser à une situation qui illustre comment certaines barrières ont pu empêcher la prestation de soins de soutien?

**Section 3. Perceptions des facilitateurs**

1. Quels sont, à votre avis, les principaux facilitateurs des mesures de soutien optimales pour les patients dans les unités de traitement du virus Ebola? Y a-t-il d’autres éléments facilitants que vous identifiez?
2. Comment sont-ils liés aux ressources matérielles, aux fournitures et à la technologie? Pourriez-vous expliquer?
3. Comment sont-ils liés aux ressources humaines pour la gestion clinique des patients admis dans les unités de traitement du virus Ebola?
4. Comment sont-ils liés à la structure organisationnelle de la prise en charge clinique?
5. Pouvez-vous penser à une journée ou les choses ont bien fonctionnées? Pourriez-vous partager avec nous une situation qui illustre la façon dont certains facilitateurs ont pu améliorer la prestation des soins de soutien?

**Section 4. Mesures de référence**

1. Dans le contexte que vous avez connu lors de l’épidémie Ébola, à votre avis, quels sont les soins minimaux qui doit être fournis aux patients admis aux unités de traitement?
2. Est-ce que les soins minimaux que vous venez de nommer devraient être considérés comme des mesures de références ou encore des critères d’évaluation quant aux soins adéquats fournis aux patients admis dans les unités de traitement du virus Ebola ? Y en a-t-il d’autres que vous aimeriez ajouter ?’

**Section 5. Solutions, recommandations**

1. Quelles sont les solutions potentielles qui aideront à surmonter les obstacles qui entravent la prise en charge dans les unités de traitement du virus Ebola?
2. Quelles sont vos recommandations concernant la planification, la préparation et la prestation des soins aux patients admis aux unités de traitement du virus Ebola? Auriez-vous des recommandations?

**Section 6. Conclusions**

1. Y a t-il des éléments que nous n’avons pas discutés et qui semblent pertinents à mentionner sur la prise en charge clinique dans les unités de traitement du virus Ebola?
